# eDiVA—Classification and prioritization of pathogenic variants for clinical diagnostics

**DOI:** 10.1002/humu.23772

**Published:** 2019-05-21

**Authors:** Mattia Bosio, Oliver Drechsel, Rubayte Rahman, Francesc Muyas, Raquel Rabionet, Daniela Bezdan, Laura Domenech Salgado, Hyun Hor, Jean‐Jacques Schott, Francina Munell, Roger Colobran, Alfons Macaya, Xavier Estivill, Stephan Ossowski

**Affiliations:** ^1^ Centre for Genomic Regulation (CRG) The Barcelona Institute of Science and Technology Barcelona Spain; ^2^ Universitat Pompeu Fabra (UPF) Barcelona Spain; ^3^ Barcelona Supercomputing Center (BSC) Barcelona Spain; ^4^ Bioinformatics Unit (MF1), Department for Methods Development and Research Infrastructure Robert Koch Institute Berlin Germany; ^5^ NKI Netherlands Cancer Institute The Netherlands; ^6^ Institut de Recerca Sant Joan de Déu University of Barcelona Barcelona Spain; ^7^ Department of Neurology University Hospital Zurich Zurich Switzerland; ^8^ L'Institut du Thorax, INSERM, CNRS Univ Nantes Nantes France; ^9^ Service de Cardiologie, L'institut du thorax CHU Nantes Nantes France; ^10^ Vall d'Hebron Institut de Recerca (VHIR) Barcelona Spain; ^11^ Sidra Medicine Doha Qatar; ^12^ Women's Health Dexeus Barcelona Spain; ^13^ Institute of Medical Genetics and Applied Genomics University of Tübingen Tübingen Germany

**Keywords:** disease variant prioritization, machine learning, NGS diagnostics, rare genetic disease, whole‐exome sequencing

## Abstract

Mendelian diseases have shown to be an and efficient model for connecting genotypes to phenotypes and for elucidating the function of genes. Whole‐exome sequencing (WES) accelerated the study of rare Mendelian diseases in families, allowing for directly pinpointing rare causal mutations in genic regions without the need for linkage analysis. However, the low diagnostic rates of 20–30% reported for multiple WES disease studies point to the need for improved variant pathogenicity classification and causal variant prioritization methods. Here, we present the exome Disease Variant Analysis (eDiVA; http://ediva.crg.eu), an automated computational framework for identification of causal genetic variants (coding/splicing single‐nucleotide variants and small insertions and deletions) for rare diseases using WES of families or parent–child trios. eDiVA combines next‐generation sequencing data analysis, comprehensive functional annotation, and causal variant prioritization optimized for familial genetic disease studies. eDiVA features a machine learning‐based variant pathogenicity predictor combining various genomic and evolutionary signatures. Clinical information, such as disease phenotype or mode of inheritance, is incorporated to improve the precision of the prioritization algorithm. Benchmarking against state‐of‐the‐art competitors demonstrates that eDiVA consistently performed as a good or better than existing approach in terms of detection rate and precision. Moreover, we applied eDiVA to several familial disease cases to demonstrate its clinical applicability.

## 1 INTRODUCTION

Rare genetic diseases are classical models for studying gene function and linking genotypes to disease phenotypes. Although each of these diseases only affects a small number of patients, the sum of people affected by one of the more than 7,000 rare diseases exceeds 30 million individuals in the US alone (Cutting, 2014). Whole‐exome sequencing (WES), and more recently whole‐genome sequencing (WGS), are routinely applied to identify variants causing rare Mendelian diseases in studies of families or parent–child trios (Choi et al., 2009; Ng et al., 2010; Louis‐Dit‐Picard et al., 2012; Rabbani, Mahdieh, Hosomichi, Nakaoka, & Inoue, 2012).

Usually, each exome sequencing experiment yields tens of thousands of genetic variants in coding and splicing regions that require thorough functional annotation and filtering to allow identification of the causal variant. Several tools have been published performing variant annotation, including Annovar, VEP, or SNPeff, which augment the sequencing information with a comprehensive set of current omics, population genomics, and clinical knowledge (Cingolani et al., 2012; McLaren et al., 2016; Wang, Li, & Hakonarson, 2010). These tools utilize a large selection of available databases containing gene annotations, various genomic features, variant allele frequencies in different populations, functional impact prediction, and evolutionary conservation (Bao et al., 2014). Other methods, such as eXtasy (Sifrim et al., 2013), PhenoDB (Sobreira, Schiettecatte, Boehm, Valle, & Hamosh, 2015), Phen–Gen (Javed, Agrawal, & Ng, 2014), VarSifter (Teer, Green, Mullikin, & Biesecker, 2012), KGGseq (M.‐X. Li, Gui, Kwan, Bao, & Sham, 2012), and SPRING (Wu, Li, & Jiang, 2014), focus on prioritization of potentially causal variants using both functional annotation and clinical information. These tools systematically filter, evaluate, and prioritize thousands of variants, taking into account knowledge found in genome annotation databases (Rhead et al., 2010), disease gene repositories (OMIM, Online Mendelian Inheritance in Man; Landrum et al., 2014), and patient pedigree information, as well as phenotype descriptions and disease definitions provided for example, as Human Phenotype Ontology (HPO) terms (Köhler et al., 2014). Finally, methods such as Endeavour (Tranchevent et al., 2008) and GeneDistiller (Seelow et al., 2008) prioritize disease genes, not individual variants, by integrating diverse genomic data sources.

Detection rates of causal variants using WES have been reported to be as low as 20–30% of cases (H. Lee et al., 2014; Yang et al., 2013), although higher success rates have been reported for specific disease or inheritance types (Sawyer et al., 2016) and for studies using parent–child trios (Yang et al., 2013). While some of the unsolved cases might be explained by intergenic or intronic regulatory variation or unidentified structural variants, the low detection rate also indicates the need for development of better prioritization strategies for coding variants and robust classifiers comprehensively integrating the available amount of prior omics and the knowledge of the disease.

Many computational algorithms have been developed to assess pathogenicity of genetic variants. Tools such as SIFT (Kumar, Henikoff, & Ng, 2009), CADD (Kircher et al., 2014), PolyPhen‐2 (Adzhubei et al., 2010), or Eigen (Ionita‐Laza, McCallum, Xu, & Buxbaum, 2016) are commonly used in clinical practice to help variant interpretation. They derive a functional impact score based on amino acid or nucleotide conservation, and biochemical properties of the amino acid changes as features. While some algorithms additionally categorize variants into various categories such as “neutral,” “benign,” “deleterious,” “damaging,” “probably‐damaging,” or “pathogenic” (e.g., SIFT, Condel, PolyPhen‐2, and Mutation Assessor), scores of other methods need to be interpreted by using (often arbitrary) cutoffs for pathogenicity (e.g., CADD). These predicted pathogenicity labels are an integral part of the American College of Medical Genetics and Genomics standards and guidelines for the interpretation of sequence variants (Richards et al., 2015). Methods combining multiple classifiers, such as MetaLR, have been shown to produce better results than single classifiers (Dong et al., 2015). Recently, specialized ensemble learning methods for estimating pathogenicity of rare variants have been published: Mendelian Clinically Applicable Pathogenicity (M‐CAP; Jagadeesh et al., 2016), using gradient‐boosting trees on pathogenicity scores and conservation features, and Revel (Ioannidis et al., 2016), using an RF to integrate several pathogenicity predictors.

To combine an intuitive user interface with comprehensive variant prediction, annotation, pathogenicity classification, and causal variant prioritization we developed eDiVA (exome Disease Variant Analysis), http://www.ediva.crg.eu. The eDiVA pipeline is composed of four main components: (a) eDiVA‐Predict, where sequencing results are processed to predict the presence of genomic variants; (b) eDiVA‐Annotate, that enriches variants via a domain‐knowledge database; (c) eDiVA‐Score, which estimates variant pathogenicity using a random forest model; and (d) eDiVA‐Prioritize, in which variants from small groups of related samples (i.e., families or parent–child trios) are analyzed jointly. eDiVA returns a shortlist of candidate variants compatible with the selected disease inheritance model and the pedigree information. Using the pathogenicity probability computed by eDiVA‐Score, variants are ranked such that better candidates appear on top of the result list. eDiVA has been developed with specific emphasis on usability, automation, and reproducibility of results and is available as a web service with a graphical user interface (see Supporting Information Material), or as an open‐source repository with Docker containers. eDiVA can be run using the NextFlow (Di Tommaso et al., 2017) pipeline management system to ensure its compatibility with most standalone or cloud‐computing platforms as well as to guarantee reproducibility on any system.

eDiVA has been optimized for two common clinical diagnostics scenarios, parent–child trios comprised of healthy parents and one affected child (tested for recessive, compound heterozygous, and X‐linked inheritance or dominant de novo variants) and families with multiple affected relatives (additionally tested for dominant inheritance). We demonstrate that eDiVA outperforms competing approaches in a semisynthetic benchmark study introducing thousands of known disease variants from ClinVar (Landrum et al., 2014) or HGMD (Stenson et al., 2017) into real WES data from the 1000 Genomes Project CEPH parent–offspring trio of European ancestry (NA12878, NA12891, and NA12892). We, furthermore, report summary statistics on eDiVA and Phen–Gen results for 35 unreported disease cases, composed of 15 cases of spinocerebellar ataxia, 16 cases of primary immunodeficiency, and four cases of congenital myasthenia.

## 2 MATERIALS AND METHODS

### 2.1 eDiVA pipeline

eDiVA consists of a Python pipeline combined with an SQL Database back‐end composed of four components: variant prediction, variant annotation, pathogenicity estimation, and variant prioritization (Figure S1). The main functionality of eDiVA is to process next‐generation sequencing (NGS) data for small sets of samples (e.g., families or parent–child trios) and to output a shortlist of potentially causal variants for the diagnosed disease. eDiVA is available as an open‐source repository, https://github.com/mbosio85/ediva, with a Docker container composition wrapped within a NextFlow (Di Tommaso et al., 2017) interface to guarantee exact reproducibility on the most common computing platforms (including several cloud platforms) and as a freely accessible web server: http://www.eDiVA.crg.eu. The modular nature of eDiVA allows for easy integration of specific parts, for example, the eDiVA‐Score module for pathogenicity estimation, in other pipelines or tools. Comprehensive examples for the use of eDiVA and example input files are included in the repository and on the website.

### 2.2 eDiVA‐Predict: WES or WGS processing and variant calling

The eDiVA‐Predict module performs sample‐wise variant calling according to the recent GATK (McKenna et al., 2010) best practices (https://www.broadinstitute.org/gatk/guide/best‐practices as of June 2017) to extract genetic variants from raw reads. Reads in fastq format are aligned using bwa‐mem (H. Li, 2013), alignments are post‐processed using samtools (H. Li, 2011), GATK (McKenna et al., 2010), Picard (Picard Tools—By Broad Institute), and custom quality filters (details provided in Supporting Information Material). Finally, VCF files are generated using GATK HaplotypeCaller. Subsequent regenotyping of all positions harboring a single‐nucleotide variant (SNV) or small insertions and deletions in at least one family member yields a complete matrix of variants for the whole sample set (family) in multisample VCF format. Due to the computational resources required for read alignment and variant calling, eDiVA‐Predict is currently not enabled on the eDiVA web server, but can be used with the standalone version of eDiVA on a local or remote computing infrastructure (e.g., Amazon Cloud). Alternatively, variant prediction can be performed using any tool able to produce one multisample VCF file reporting genotype quality and coverage information for all variable positions (e.g., GATK, McKenna et al., 2010; freebayes, Garrison & Marth, 2012).

### 2.3 eDiVA‐Annotate: Functional variant annotation

Using the eDiVA‐Annotate module each variant is individually linked with public information sources to integrate multiple knowledge domains, and to provide a comprehensive annotation profile. First, ANNOVAR (Wang et al., 2010) is applied to relate each variant to its corresponding gene (choosing among UCSC, Ensembl, or Refseq gene annotations), and to its functional consequence at the protein level. Next, functional, population genomics, and evolutionary data relevant for variant prioritization are added to each variant. To this end we created a MySQL database, eDiVA‐DB, containing all relevant positional information obtained from UCSC table browser (Rhead et al., 2010) and other sources. Each variant is annotated with population allele frequency information from the dbSNP (Sherry et al., 2001), discovEHR (Dewey et al., 2016), 1000 Genomes Project (1000GP; The 2015 Genomes Project Consortium, 2015), Exome Variant Server (Exome Variant [Link]), and GnomAD exomes (Lek et al., 2016) databases. The latter three databases also provide information on specific populations (e.g., Caucasian, Asian, African American, etc.), which can be selected for improved causal variant prioritization. Information on evolutionary conservation is incorporated from PhyloP (Rhead et al., 2010), and PhastCons (Hubisz, Pollard, & Siepel, 2011), including conservation scores for primates, mammals, and vertebrates. Precalculated scores for functional impact of variants have been integrated based on the algorithms SIFT (Kumar, Henikoff, & Ng, 2009), PolyPhen‐2 (Adzhubei et al., 2010), Mutation Assessor (Reva, Antipin, & Sander, 2011), Condel (González‐Pérez & López‐Bigas, 2011), Eigen (Ionita‐Laza et al., 2016), and CADD (Kircher et al., 2014). Furthermore, eDiVA‐DB includes information on genomic features like segmental duplications and simple sequence repeats provided by UCSC table browser (Rhead et al., 2010). Finally, eDiVA‐DB provides clinical data from ClinVar (Landrum et al., 2014) and OMIM related to each variant and affected gene.

eDiVA‐Annotate uses multisample VCF files and returns a file with annotated variants in comma‐separated value format. This step can be performed on the eDiVA web server.

### 2.4 eDiVA‐Score: Estimating variant pathogenicity

**Figure 1:**
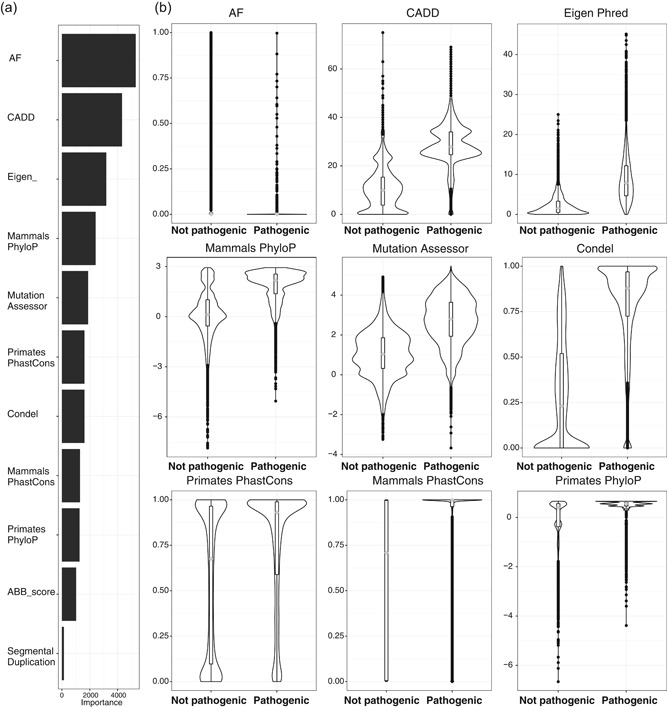
eDiVA‐Score random forest model. (a) Estimated importance of features used in the model (extracted with varImp command). (b) Distribution of values for the top‐9 features used in the model, comparing ClinVar pathogenic against ClinVar nonpathogenic variants. AF: allele frequency; eDiVA: exome Disease Variant Analysis

eDiVA's prioritization algorithm relies on accurate estimation of pathogenicity for each variant. We therefore developed eDiVA‐Score, a machine learning classifier, which assigns a pathogenicity probability to each variant based on its annotation characteristics obtained from eDiVA‐Annotate. eDiVA‐Score is built by training a random forest (RF) model using the R “randomForest” package with 1000 binary classification trees (Breiman, 2001; Hastie, Tibshirani, & Friedman, 2009) and five‐fold cross validation. Eleven features were selected to train the RF model: (a) the maximum minor allele frequency (MAF) of 1000Genomes and GnomAD databases; (b) four conservation measures (conservation in primates and mammals using the PhastCons (Hubisz et al., 2011) and PhyloP (Pollard, Hubisz, Rosenbloom, & Siepel, 2010); (c) four functional impact predictors: Condel (González‐Pérez & López‐Bigas, 2011), Phred‐scaled CADD score (Kircher et al., 2014), Eigen (Ionita‐Laza et al., 2016), and Mutation Assessor (Reva et al., 2011); (d) the likelihood to be in a segmental duplication, which correlates with false‐positive variant calls (Ho, Tsai, Chen, & Lin, 2011); and (e) an in‐house estimator of systematic sequencing errors called ABB‐score (Muyas et al., 2019). Note that Condel, Eigen and CADD are combination scores integrating several features also included in eDiVA‐score, namely evolutionary conservation (PhastCons and PhyloP in mammals and primates) and Mutation Assessor scores. The RF model has been trained using 15,000 random pathogenic and likely pathogenic variants from the ClinVar database (Landrum et al., 2014) as positive cases. We then built a control set composed of 15,000 nonpathogenic variants from ClinVar, and 100,000 random variants from GnomAD (Lek et al., 2016) not contained in ClinVar. The vast majority of variants in both positive and negative training set are rare (allele frequency [AF], <1%; Figure S2a,b), thus circumventing that AF dominates the classification model. Following the neutral theory of molecular evolution (Kimura, 1983) missing data is generated using expected values for nonpathogenic (neutral) variants (Figure 1). The only exception is AF, as missing data in the context of AF means that the SNV is novel, that is, has AF of zero. Variants used for training of the RF have been excluded in all benchmarking tests performed in this study.

### 2.5 eDiVA‐Prioritize: Causal variant prioritization

Causal variant prioritization consists of four steps, (a) ranking by estimated probability of variants to cause a phenotypic change (eDiVA‐Score, see above); (b) removal of all variants that do not segregate according to the selected inheritance mode; (c) filtering based on functional and population genomic features; and (d) prioritization based on user defined clinical phenotypes (as HPO IDs). Filtering based on segregation requires the user to submit a simple pedigree file defining the relationship between samples and their disease state (i.e., affected or unaffected), and to choose the most likely inheritance pattern for the disease (or to run all modes). eDiVA‐Prioritize can process variants following five types of inheritance patterns: (a) dominant de novo, (b) autosomal dominant inherited, (c) autosomal recessive homozygous, (d) autosomal recessive compound heterozygous, or (e) X‐linked.

Optionally, eDiVA removes variants that are improbable of being damaging, are likely false‐positive calls or do not have sufficient read coverage in all family members to reliably estimate segregation patterns. By default, eDiVA applies a lenient filter setting defined in Table S1. Finally, eDiVA allows the user to specify a list of HPO terms (Köhler et al., 2014) relevant for the disease as an additional source of information to prioritize variants in genes. eDiVA highlights all variants in genes related to the submitted phenotypic traits using a custom algorithm to estimate the HPO‐gene association (detailed in the Supporting Information material).

### 2.6 Performance evaluation using semisynthetic cases

To assess the performance of eDiVA and several competing methods, we implemented a semisynthetic benchmark based on real WES data from a trio in which we spiked‐in known pathologic variants from the ClinVar database (Landrum et al., 2014). We chose a publicly available CEPH trio sequenced within the framework of the 1000 Genomes Project composed of samples with European ancestry NA12878 (daughter), NA12891 and NA12892 (parents), downloadable from https://public_docs.crg.es/sossowski/MicrobeGenomes/human/eDiVA/insilico_simulation_data/, and we called variants and generated a multisample VCF file using eDiVA‐Predict. For the purpose of this benchmark study, all 138,705 variants found in the original trio are considered true negatives, that is, variants not associated with the disease.

Next, we embedded known disease variants in the trio following segregation patterns expected for Mendelian diseases. This positive set, containing variants associated with the diseases, consists of all variants from ClinVar (Landrum et al., 2014) database labeled as “pathogenic” or “likely pathogenic”, having an OMIM reference in the database and that had not been used for training of eDiVA‐Score. For each pathogenic variant, we extracted: chromosome, position, reference and alternative nucleotides, dbSNP identifier, gene name, inheritance mode of the associated disease (where available, randomly assigned otherwise), and HPO terms for the disease. Variants without HPO annotation have been excluded from the benchmark set.

We have simulated three inheritance patterns: autosomal recessive homozygous, autosomal recessive compound heterozygous, and dominant de novo, as these are the most likely patterns found in parent–child trio based rare‐disease diagnostics. To create realistic disease genotypes, each pathogenic variant was introduced into the exomes of the daughter and the parents, if applicable according to the inheritance mode. The read distribution of reference and alternative reads was simulated depending on the inheritance mode and the original coverage data. The variant allele frequency (VAF) of the alternative allele (i.e., the fraction of reads showing the alternative allele) introduced in the original VCF file has been obtained using a beta distribution and a binomial distribution for homozygous and heterozygous variants, respectively. A total of 6,811 disease‐associated variants from ClinVar not previously used in the training of eDiVA‐Score were used for benchmarking: 3,353 recessive homozygous, 2,592 dominant de novo, and 866 recessive compound heterozygous disease‐causing variants (see Table S2 for additional information on simulated genotypes).

### 2.7 Benchmarking of variant pathogenicity estimation methods

We evaluated the ability of eDiVA‐Score and six competing methods, namely CADD, Eigen, DANN, Revel, M‐CAP, and MetaLR (Dong et al., 2015, Ioannidis et al., 2016, Ionita‐Laza et al., 2016, Jagadeesh et al., 2016, Kircher et al., 2014, Quang, Chen, & Xie, 2015), to prioritize pathogenic over benign variants. We generated a receiver operating characteristic (ROC) curve for each tool and benchmark set and measured performance by area under the curve (AUC).

**Figure 2:**
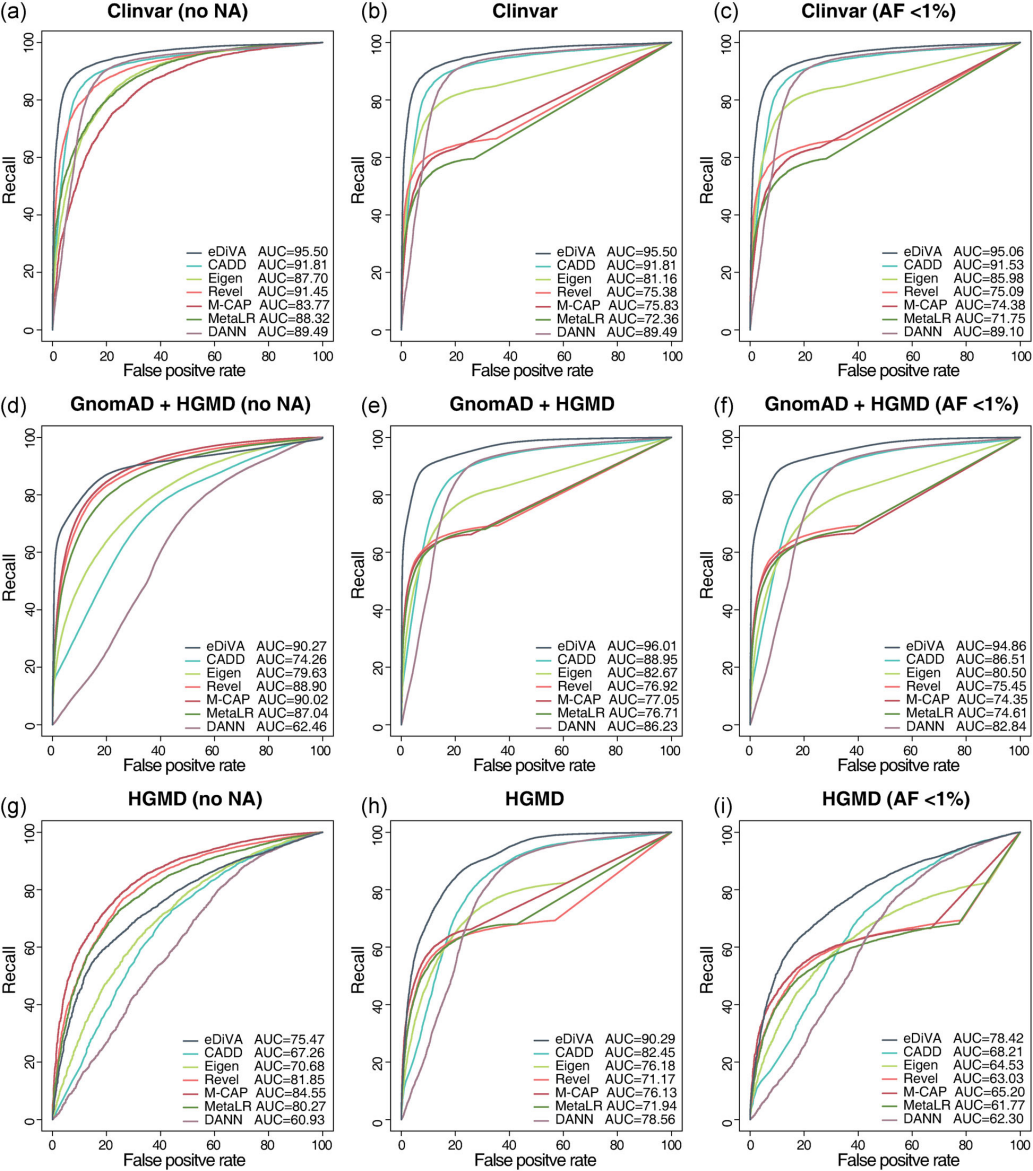
Benchmarking of the pathogenicity classifiers eDiVA‐Score, CADD, Eigen, Revel, and M‐CAP using ROC for (a) set of 10,494 ClinVar pathogenic variants (TP) and 3,887 ClinVar “benign” variants (TN); (b) set of 16,694 ClinVar pathogenic variants (TP) and 19,888 ClinVar “benign” variants (TN), setting missing values to benign, (c) subset of rare variants (AF, <1% from set c); (d) set of 63,712 variants from HGMD (TP) and 100,000 from GnomAD (TN) for which values from all tools are available; (e) set of 96,569 variants from HGMD (TP) and 100,000 from GnomAD (TN), setting missing values to benign; (f) subset of rare variants (AF, <1% from set e); (g) set of 63,712 HGMD variants (“DM” and “DM?”) as TP, and 1,892 HGMD variants (other categories) as TN for which values from all tools are available; (h) set of 96,569 variants from HGMD (“DM” and “DM?”) as TP, and 7,376 HGMD (other categories) as TN, setting missing values to benign; and (i) subset of rare variants (AF, <1% from set h). AF: allele frequency; eDiVA: exome Disease Variant Analysis; M‐CAP: Mendelian clinically applicable pathogenicity; ROC: receiver operating characteristic; TN: true negative; TP: true positive

**Figure 3:**
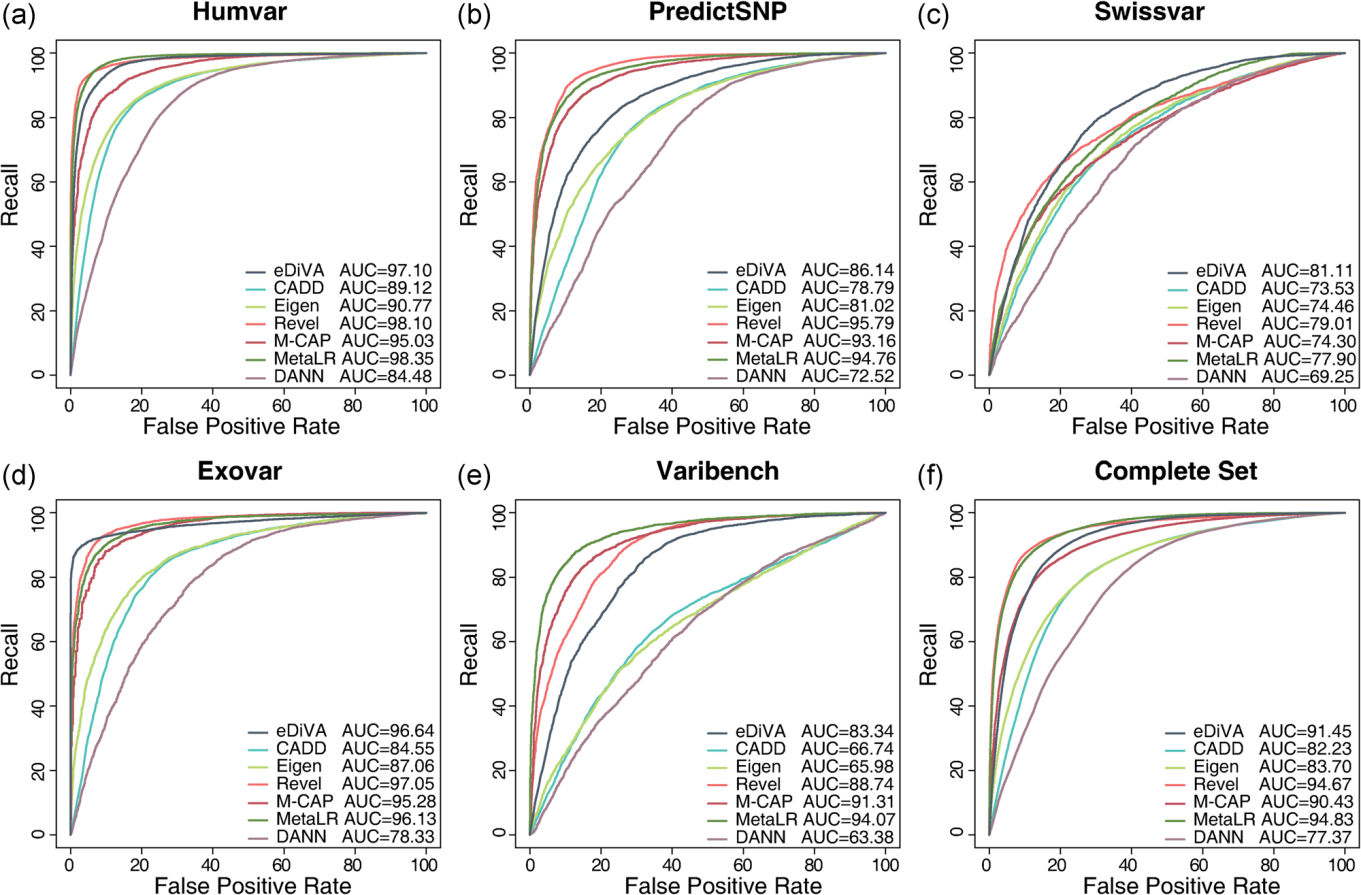
Receiver operating characteristic curves comparing pathogenicity classifiers on five independent data sets (and the combined set) composed of pathogenic and neutral variants. Revel, M‐CAP, and eDiVA show a similarly strong performance, with the exception of the PredictSNP and Varibench sets, on which Revel and M‐CAP outperform eDiVA‐Score. eDiVA: exome Disease Variant Analysis; M‐CAP: Mendelian clinically applicable pathogenicity; SNP: single‐nucleotide polymorphism

First, we evaluated the performance of each method on the ClinVar test set (containing only variants not used for model training), using variants labeled “pathogenic” as true positives (TP) and variants labeled “benign” as true negatives (TN; Figure 2a–c). Second, we benchmarked using variants from the HGMD and GnomAD databases (not used in model training or present in ClinVar) as TP and TN, respectively (Figure 2d–f). Third, we measured the performance of all methods on HGMD data only, using the categories for damaging and likely damaging mutation (DM and DM?) as TP and any other HGMD category as false positives (FP) (Figure 3g–i). Functional impact values for the benchmarked methods have been obtained from the respective publications. CADD, DANN, and eDiVA provide damage estimates for all positions of the genome, and Eigen for close to 70% of all positions, whereas Revel, M‐CAP, and MetaLR are trained specifically for rare (AF, <1%) or known variants and are only available for a subset of ClinVar, HGMD, and GnomAD. We, therefore, performed three separate performance tests for each of the three benchmark sets, applying the following criteria (a) using only variants having Revel and M‐CAP scores available (ClinVar: 3,887 TP and 10,494 TN; HGMD/GnomAD: 63,712 TP and 100,000 TN; HGMD: 63,712 TP and 1,892 TN); (b) random subset of all variants, assigning a default value of 0 to missing values (ClinVar: 19,888 TP and 16,694 TN; HGMD/GnomAD: 96,569 TP and 100,000 TN; HGMD: 96,569 TP and 7376 TN); and (c) using only rare variants (AF, ≤0.01) from the previous pool of variants (ClinVar: 16,531 TP and 15,531; HGMD/GnomAD: 90,004 TP and 97,828 TN; HGMD: 96,004 TP and 2,817 TN).

Furthermore, we studied five variant sets provided by Grimm et al. (2015), forming a collection of data sets for benchmarking pathogenicity classifiers published in independent studies. Finally, we combined these five sets to form a combined benchmark (see Supporting Information Materials for details).

### 2.8 Benchmarking of disease variant prioritization methods

We compared eDiVA with three commonly used tools for variant annotation and prioritization: Exomiser (Robinson et al., 2014), PhenoDB (Sobreira et al., 2015), and Phen–Gen (Javed et al., 2014) on a set of 6,811 semisynthetic parent–child trios (see above). PhenoDB was executed from the https://phenodb.org/ website using standard parameters (a) AF, <0.01; (b) including variants which are present in dbSNP, and (c) analysis type chosen among “autosomal recessive compound heterozygous”, “autosomal recessive homozygous”, or “autosomal dominant new mutation”. We locally installed Phen–Gen and launched it with the corresponding setups: (a) “Recessive”, “allow_de_novo = 0” for recessive and compound inheritance, and (b) “Dominant”, “allow_de_novo = 1” for the dominant de novo inheritance model. We locally installed Exomiser and analyzed all trio cases using PhenIX prioritization mode (details in Supporting Information Material). We tested eDiVA in two configurations, (a) without phenotype description, and (b) using HPO IDs describing the disease phenotype for disease‐specific prioritization of candidate variants.

To benchmark the ability of eDiVA, Exomiser, PhenoDB, and Phen–Gen to distinguish disease‐causing from benign variants we compared three quality metrics, (a) recall (i.e., did the causal variant appear in the output list or not), (b) average number of false positives across all benchmarked cases as a proxy for precision, and (c) ranks of causal variants reported for each mode of inheritance using violin plots (Figure 3a–c). To compare ranks, variants reported by eDiVA are sorted by eDiVA‐Score, Phen–Gen results are sorted by DCOD‐score (“Probability of deleteriousness based on genic predictor”), and Exomiser results are sorted by “Exomiser Gene Combined Score”. Results of PhenoDB are presented in the default order (chromosome and position), as no prioritization score is provided.

## 3 RESULTS

### 3.1 eDiVA: A platform for pathogenicity estimation and causal variant prioritization

eDiVA is a disease variant prioritization tool optimized for NGS‐based genetic disease diagnostics in families and parent–child trios. It is composed of four components: eDiVA‐Predict handles read alignment and variant prediction, eDiVA‐Annotate performs functional annotation of variants, eDiVA‐Score estimates the probability of variants to be pathogenic, and eDiVA‐Prioritize filters and ranks variants according to various quality criteria, proper segregation, and likelihood to cause phenotypic changes. eDiVA is available as standalone software at https://github.com/mbosio85/ediva, and as a web service providing access to functional annotation, pathogenicity classification and causal variant prioritization modules (www.ediva.crg.eu). The eDiVA web service facilitates analysis of families or parent–child trios in a few clicks, requiring only a VCF file, and optionally a set of HPO IDs describing the disease phenotype. eDiVA returns a shortlist of candidate variants and genes, ranked by pathogenicity score (together with gene relatedness to the specified HPO IDs if available), and including all annotation features in comma‐separated value (.csv) and Microsoft Excel (.xlsx) format.

### 3.2 Benchmarking eDiVA and competing methods

To comprehensively evaluate eDiVA's performance in finding disease‐causing variants, and to compare it to previously published tools, we performed a benchmark in two categories. First, we evaluated the ability of eDiVA‐Score to distinguish disease‐causing from benign variants compared to four publicly available methods for estimating deleteriousness. Second, we benchmarked the performance of eDiVA, PhenoDB, and Phen–Gen on identification of causal variants using semisynthetic parent–child trios analyzed by WES, optionally allowing for the use of clinical phenotype descriptions for causal variant prioritization.

### 3.3 Benchmarking of eDiVA‐Score and other variant pathogenicity classifiers

We developed eDiVA‐Score, a machine learning‐based method for estimating variant pathogenicity (deleteriousness) independent of any prior clinical information (see Section 2). Feature‐selection identified population allele frequency, functional impact, and conservation in placental mammals as the most important features (Figure 1a). The correlation matrix for all features is shown in Figure S3. Features selected for inclusion in the RF show distinct distributions for pathogenic variants compared to benign variants in ClinVar (Figure 1b), random coding variants reported in GnomAD (Figure S4b). All integrated conservation scores (PhyloP and PhastCons scores for vertebrates, mammals and primates) classify pathogenic variants better than random, but perform worse than any specialized method for estimating functional impact or pathogenicity (Figure S5).

We benchmarked the ability of eDiVA‐Score, CADD, DANN, Eigen, Revel, M‐CAP, and MetaLR to predict the deleteriousness of variants and to distinguish pathogenic from benign variants in nine setups (Section 2). We first compared the performance on classifying pathogenic and benign variants from ClinVar (Figure 2a), on distinguishing disease variants from HGMD (Stenson et al., 2017) from 100,000 random variants from GnomAD (Figure 2d), for which scores are available for all methods. Note that Revel and M‐CAP have been trained on a subset of the HGMD variants (e.g., using class “DM” as positive training set), giving them an advantage due to potential overfitting in any of the following benchmark tests using HGMD variants (for an in‐depth discussion of the interplay between overfitting and circularity in training and benchmarking data (Grimm et al., 2015). Using ROC analysis, we found that eDiVA‐Score distinguishes disease‐causing and benign variants with high sensitivity and recall in both benchmark sets (AUC of 0.95 and 0.90), considerably better than CADD (AUC of 0.91 and 0.74), DANN (AUC of 0.89 and 0.82), Eigen (AUC of 0.87 and 0.77), Revel (AUC of 0.91 and 0.89), M‐CAP (AUC of 0.84 and 0.90), and MetaLR (AUC of 0.88 and 0.87). Of note, eDiVA‐Score showed better precision‐recall curves than competing methods (Figure S6).

Disease variant prioritization tools depend on pathogenicity values for any position of the exome, since de novo mutations can occur randomly and novel ultra‐rare variants are still being discovered. Therefore, we next benchmarked the methods on random variants chosen from the complete ClinVar and HGMD/GnomAD benchmark sets, whereas setting missing data to benign (Section 2). As expected, the recall of Revel, M‐CAP, and MetaLR decreased substantially due to missing information, whereas the other methods performed slightly better than in the previous tests (Figure 2b,e). Finally, we tested how the methods perform on classification of rare variants (AF, <0.01), otherwise following the same criteria for selection of the test sets as in the previous benchmark (Figure 2c,f). Again, eDiVA‐Score shows the best performance of all methods.

We wondered if the use of random GnomAD variants as TN (nonpathogenic) set might bias the results of the HGMD/GnomAD benchmark due to for example, overfitting onto the allele frequency feature. Therefore, we next measured the performance of all methods on HGMD data only, using the categories for highly likely pathogenic (“DM” and “DM?”) as TP set and less likely pathogenic (any other HGMD category) as TN set (Section 2). We performed the same three tests as discussed above for the ClinVar and HGMD/GnomAD benchmark sets. On the subset of variants for which scores are available for all methods (Figure 2g) eDiVA's performance (AUC 0.77) was found to be slightly lower than MetaLR's (0.80), Revel's (AUC 0.82), and M‐CAP's (AUC 0.85), but substantially better than the performance of the other general‐purpose methods CADD (AUC 0.67) and Eigen (AUC of 0.70). However, eDiVA still outperformed all other methods on the complete HGMD variant set (missing scores set to benign), as well as on the rare variant set (Figure 2h,i).

Finally, we compared the performance of all methods on a benchmark set compiled by Grimm et al. (2015), consisting of mutually exclusive subsets of the previously published benchmark sets Varibench, HumVar, ExoVar, predictSNP, and SwissVar (see Supporting Information Material for details). These popular benchmark data sets differ in the way they define pathogenic and neutral variants, for example, the maximum AF for pathogenic variants can differ dramatically, allowing us to benchmark diverse challenges. Furthermore, Grimm et al. filtered these benchmark sets to minimize overlap between them, reducing the likelihood that tools are benchmarked on variants they have been trained on and hence reducing the impact of overfitting on the benchmark results (Grimm et al., 2015). We found that none of the methods consistently performs better than other methods, but that eDiVA‐Score, M‐CAP, Revel, and MetaLR show comparably high performance, except on PredictSNP and Varibench, for which MetaLR, Revel, and M‐CAP show a better performance than eDiVA‐Score. PredictSNP incorporates HGMD variants in the positive and negative control sets; see Tables 2 and 3 of Grimm et al. (2015), likely to be giving a strong advantage to Revel and M‐CAP, which have been trained on HGMD. CADD, DANN, and Eigen performed significantly worse than the other three methods on all benchmark sets. Note that CADD, DANN, Eigen, and MetaLR have been trained to predict deleteriousness (or more general the functional impact) of variants, whereas eDiVA‐Score, Revel, and M‐CAP have been trained to identify pathogenic variants, partly explaining the divergent performance levels across the different benchmark sets. Moreover, eDiVA‐score, MetaLR, and M‐CAP use CADD as one of many features, explaining the better performance of the derived scores.

In summary, our benchmark results demonstrate the good performance of eDiVA‐Score as pathogenicity classifier, comparable to and often better than state‐of‐the‐art methods available to date. Furthermore, eDiVA‐Score outperforms other general‐purpose methods not restricted by variant AF (i.e. CADD, DANN, and Eigen), while showing competitive results when compared with specialized tools such as MetaLR, M‐CAP, and Revel, which are only available for known (rare) SNVs.

### 3.4 Causal variant prioritization in parent–child trios

We benchmarked the performance of eDiVA and three widely used tools, PhenoDB, Phen–Gen, and Exomiser, on identification of causal variants for rare Mendelian diseases in parent–child trios. To this end, we simulated three scenarios typically encountered in parent–child trio diagnostics, (a) autosomal dominant de novo, (b) autosomal recessive homozygous, and (c) autosomal recessive compound heterozygous Mendelian inheritance modes. In total, we simulated 6,811 semisynthetic parent–child trios by integrating reported pathogenic variants from ClinVar into real WES data of a trio obtained from 1000GP (see Section 2 and Table S2).

**Figure 4:**
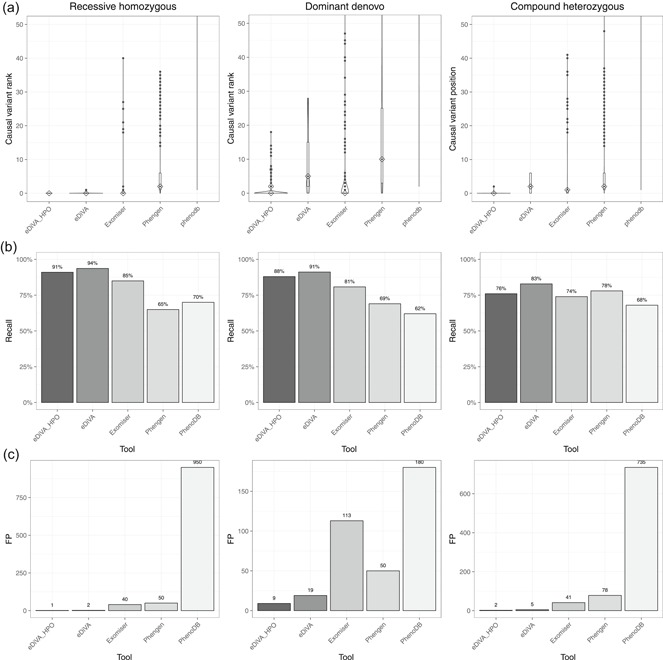
Benchmark of the causal variant prioritization tools eDiVA, Exomiser, Phen–Gen, and PhenoDB. (a) Violin plots showing the rank of disease‐causing variants within the reported candidate lists for the three tested inheritance types: “recessive homozygous”, “compound heterozygous”, and “dominant de novo”; (b) Recall values for 6,811 semisynthetic trio cases, representing the fraction of identified causal variants (i.e., “solved cases”). (c) Average number of false positives reported per case as a proxy for precision. eDiVA has been tested in two configurations, with HPO‐based gene prioritization (eDiVA_HPO) and with the default configuration not using HPO terms (eDiVA). Adding HPO filtering reduces false positives at the cost of a slightly reduced Recall. HPO: Human Phenotype Ontology

Figure 3a shows violin plots with the rank distribution of causal variants in the output lists of 6,811 analyzed trios. The optimal result is a skewed distribution close to zero, meaning that the causal variant is reported as first or very close to the top of the list in the majority of cases. Here, comparison with PhenoDB is not meaningful, as PhenoDB (unlike Phen–Gen, Exomiser, and eDiVA), offers no ranking based on pathogenicity scores (but sorts by chromosome and position). Compared to Exomiser and Phen–Gen, eDiVA's ranking method shows the best performance for recessive homozygous inheritance, eDiVA and Exomiser show best performance for dominant de novo inheritance, and all tools show similarly good performance for compound heterozygous inheritance. eDiVA consistently reported causal recessive homozygous variants and compound heterozygous variants within the top five candidates (median = 1), and dominant de novo variants within the top 25 of reported candidates (median = 4; Figure 4a). Considering that the CEPH trio has been sequenced as part of the 1000GP we finally tested if the use of 1000GP allele frequency information for filtering biases the performance estimates of eDiVA. However, we found no difference when not using the 1000GP AF database (Figure S7). Nonetheless, we cannot exclude the possibility that eDiVA (or the other methods) show reduced performance in understudied populations.

The use of HPO IDs for prioritization further reduced FPs reported by eDiVA (label eDiVA‐HPO in Figure 4c). Overall, we observed a two‐fold reduction in FPs across all inheritance modes tested. However, filtering by in silico gene panels also resulted in a reduction in recall (Figure 4b,c). Finally, we observed improved prioritization ranks under all inheritance types, with the strongest impact seen for de novo variants (Figure 4a).

We also investigated the impact of incomplete or imperfect phenotyping on eDiVA's performance by altering the HPO annotation of genetic variants imported from ClinVar (see Supporting Information Methods). Benchmarking results on the semisynthetic simulation with incomplete phenotyping show a small reduction in causal gene ranking efficiency (Figure S8). However, even imperfect phenotypic information improved the performance as compared to complete exclusion of such information.

In summary, the benchmark analyses show that eDiVA achieves highly competitive causal variant prioritization performance with respect to ranking, precision and recall, while requiring no fine tuning of parameters by the user for specific inheritance types. When disease‐specific HPO term descriptors are available, eDiVA's precision is further enhanced to the point at which complete automation of causal variant identification is feasible for recessive homozygous and compound heterozygous segregation.

### 3.5 eDiVA results on clinical cases

eDiVA has successfully been used in published case studies on mitral valve prolapse (Durst et al., 2015), cystic fibrosis (Ramos et al., 2014), phenylketonuria (Trujillano et al., 2014), arthrogryposis (Wambach et al., 2017), and Opitz‐C (Urreizti et al., 2017), among others, identifying both known, as well as novel rare‐disease genes. We recently assessed the performance of eDiVA for the diagnosis of rare congenital genetic diseases using WES of 35 parent–child trios, including 15 cases of congenital ataxia, four cases of congenital myasthenia, and 16 cases of primary immunodeficiency. Here we report general statistics on the number of candidate genes per case, while case reports and novel candidate genes will be published in separate papers. Across the 35 studied trios, eDiVA on average reported a median of 11, 3, and 10 candidate genes per trio for recessive homozygous, recessive compound heterozygous, and dominant de novo inheritance mode*,* respectively, using default parameters. In comparison, Phen–Gen reported a median of 36 and 52 candidate genes for recessive (including compound heterozygous) and dominant (including de novo) inheritance mode, respectively. Histograms of reported candidate gene numbers for eDiVA and Phen–Gen are shown in Figures S9 and S10. eDiVA found causal variants in known genes for the respective disease in none cases, and variants in genes associated with closely related disease phenotypes in seven cases. Screening of Phen–Gen results did not reveal additional candidates missed by eDiVA. The function of a novel disease variant for congenital ataxia has been described in Bahamonde et al. (2015), and reports for other candidate genes are in preparation.

## 4 DISCUSSION

Despite the massive increase in sequencing capacity and the availability of highly optimized analysis tools, multiple large‐scale rare‐disease studies reported that in only 20–30% of cases a causal variant can be identified using WES. Several reasons might explain the inability of WES analysis to identify causal variants in a majority of cases, including for example, the inability to identify regulatory variants (Claussnitzer et al., 2015), our limited knowledge of the function of noncoding RNAs, generation of new exon donor or acceptor sites by intronic variants (Y. Lee et al., 2012), small copy number variations (Krumm et al., 2012), incomplete penetrance, and unknown function of coding genes, among others. However, we argue that the potential of WES has not been exhausted and that causal coding variants are often missed due to inappropriate correction of noise in the data, insufficient use of clinical (phenotypic) data, or reporting of long unranked candidate lists, requiring tedious screening by clinicians. We further claim that these shortcomings are often overlooked due to unrealistic simulated benchmark tests not reflecting the level of noise found in real family or trio NGS data.

We have addressed these problems by developing eDiVA, a pipeline that combines multisample variant calling of family data, QC and filtering, extensive functional annotation, machine learning‐based classification of deleterious variants, and prioritization of causal variants optimized for various clinical scenarios. Furthermore, we developed a highly realistic benchmark test combining real WES data of a parent–child trio with thousands of pathogenic ClinVar variants to generate 6,811 semisynthetic disease trios. Using these cases, we have demonstrated that eDiVA's pathogenicity estimator (eDiVA‐Score) as well as eDiVA's prioritization algorithm perform favorably compared to existing state‐of‐the‐art methods. eDiVA has been able to find disease‐causing variants with higher recall, fewer false positives and better ranking than competing tools in three benchmarked modes of inheritance. Finally, we evaluated the use of phenotypic descriptors for optimizing the prioritization process.

We found that adding HPO ID‐based prioritization introduces a trade‐off between recall and the number of false positives in the output list. Despite the marginal reduction in recall, focusing on known disease genes is often the preferred choice for diagnostic purposes. Our knowledge of genetic factors playing a role in disease is constantly growing, reflected in a rapid increase of genotype–phenotype relations stored in various databases. Hence, it would be beneficial to reanalyze WES data sets once in a while (e.g., every 6–12 months) to benefit from new knowledge and to facilitate identification of previously unknown/unreported causal variants. Moreover, combined reanalysis of the growing cohorts of WES data stored in many institutes would allow to identify matching causal genes across multiple families or cases. However, most analysis pipelines require substantial hands‐on time and long candidate‐variant lists have to be screened by experts, making regular reanalysis of data sets impractical. eDiVA has been developed with a specific emphasis on high reproducibility of results and complete automation of the analysis using artificial intelligence‐based methods. Machine learning classifiers are used to perform candidate ranking and prioritization, reducing hands‐on time of clinical experts to a minimum. Integration with NextFlow, moreover, guarantees reproducibility of results at later time points and on most computing platforms. Therefore, eDiVA is a dedicated solution for regular reanalysis of large disease cohorts or collections of diagnostic cases.

Additional steps can be taken to improve the identification of disease‐related variants from WES data. The availability of custom allele frequency databases with geographical specificity would help to reduce the number of false‐positive genotype–phenotype associations due to population specific variants. To this end, institutes and hospitals with access to large cohorts of sequenced exomes may use in‐house data to filter population specific variants, an approach we have pursued our self by collecting thousands of Iberian cases in an aggregated allele frequency database (http://geevs.crg.eu/, unpublished). Identification of extended homozygosity regions could in addition help to diagnose causal homozygous variants in consanguineous cases. Moreover, the integration of structural and copy number variants (SVs and CNVs) has been shown to increase recall rates substantially (Gambin et al., 2017). Despite their frequent involvement in rare diseases (McCarroll & Altshuler, 2007), CNVs are often disregarded in WES analyses, and are rarely processed in combination with point mutations. Prioritization algorithms will have to be adapted to consider compound heterozygotes composed of a point mutation in one and a CNV in the other allele. CNV analysis is currently being integrated in eDiVA and will be available in the near future.

Better use of phenotypic descriptors has the potential to improve both precision and recall of causal variant prioritization methods. We observed that HPO ID‐based prioritization dramatically improved the precision of eDiVA. However, incomplete maps of known genotype–phenotype (or gene–phenotype) relations in public databases led to a mild reduction in recall. Robinson et al. (2014) proposed a method to overcome this limitation, tapping into the genotype–phenotype associations from mouse data to solve causal variant identification for corresponding human phenotypes. Other methods based on image analysis, for example, Hadj‐Rabia et al. (2017) or face2gene (http://suite.face2gene.com/), have also shown promising results for diagnosis of patients with visible phenotypic features. Finally, an important step in the evaluation of newly discovered genotype–phenotype associations is the identification of additional cases with a similar phenotype and mutations in the same gene. Several approaches for gene matching have been published, for example, GeneMatcher (Sobreira et al., 2015), which have been connected via the Matchmaker Exchange platform. Integration of approaches using image analysis, cross‐species phenotype–genotype correlation, and gene matching has the potential to further improve AI‐based variant prioritization methods such that they can rival the diagnostic precision of clinical experts in the future.

In summary, we have shown that eDiVA is a step towards full automation of causal variant identification in family and parent–child trio data using machine learning‐based approaches. eDiVA can be used as a support tool for clinicians to find disease‐causing variants, or as a fully automated solution for periodic reanalysis of large WES (or WGS) cohorts. eDiVA is able to identify known causal disease variants with high precision and recall, and facilitates identification of novel disease variants with minimal hands‐on time.

## Supporting information

Supporting information
