# Supplementary material for: eDiVA—Classification and prioritization of pathogenic variants for clinical diagnostics
Source: Hum Mutat. 2019 May 21;40(7):865–78. doi: 10.1002/humu.23772 (PMC6767450; doi:10.1002/humu.23772)
Supplement: Supplementary file 1 — Supporting information [file HUMU-40-865-s001.pdf]

Supplemental Material

Variant calling pipeline (eDiVA-Predict)

Sample-wise analysis:

1. Read alignment with BWA mem, chimeric read filtering, and sorting by chromosome and position
2. Local realignment with GATK RealignerTargetCreator and IndelRealigner
3. Duplicate marking with Picard Markduplicates
4. Base quality recalibration with GATK BaseRecalibrator
5. Variant calling with GATK HaplotypeCaller
6. Split SNV and INDELs to be processed independently
7. Quality control for SNVs and INDELs using GATK 3.3
8. Select high quality variants and generate final call file in VCF format
    a. Filters to exclude SNPs with GATK VariantFiltration tool:
        i. clusterWindowSize 10
        ii. "MQ < 30.0 || QUAL < 25.0 "
        iii. "DP < 5 || DP > 400 || GQ < 15
    b. Filters to exclude Indels with GATK VariantFiltration tool:
        i. QD < 2.0 || FS > 200.0 || ReadPosRankSum < -20\"
        ii. "DP < 5 || DP > 400 || GQ < 15”

Multi-Sample calling for families and trios:

1. Merge the individual variant call files to obtain all variant positions across the family
2. Re-genotype all samples at all variant positions using GATK HaplotypeCaller
3. Annotate multi-sample VCF file using eDiVA-Annotate.

Comparison of pathogenicity classifiers on additional benchmark datasets:

We compared eDiVA-Score on five datasets downloaded from

http://structure.bmc.lu.se/VariBench/GrimmDatasets.php

Composed of:

- Filtered subset of HumVar (Adzhubei et al., 2010).
- Filtered subset of ExoVar (Li et al., 2013).
- Filtered subset of VariBench (Nair and Vihinen, 2013).
- Filtered subset of predictSNP (Bendl et al., 2014).
- Filtered subset of SwissVar Dec. 2014 (Mottaz et al., 2010).

We calculated the ROC curve for each dataset independently, and for the joint set of the five datasets. These datasets have been commonly used in several benchmarks. They are composed of mostly rare, non-synonymous SNPs. Each dataset defines differently the criteria to assign a variant to the pathogenic or not-pathogenic class, thus providing benchmarking set with different rules than the one used for training. We observed that M-CAP and Revel perform substantially better than in the previously described benchmarks using ClinVar or HGMD variants (Supplemental Figure 2). From Suppl. Fig. 2, we observed how scores such as Revel and M-CAP achieve better ROC than eDiVA-Score. This is justified by the fact that such scores have been expressly developed for rare SNPs, thus are better suited to discern between pathogenic and neutral variants in these subsets.

This result highlights the different fields of applicability of eDiVA-Score versus Revel or M-CAP. The first is a general-purpose score to classify all variants, without specific focus on rare nonsynonymous SNPs. Revel and M-CAP, instead focus on rare variants with impact on the amino acid sequence. It is an expected consequence, then, that Revel and M-CAP perform better on evaluation datasets close to the problem they address, rather than ranking all variants.

Exomiser benchmark parameters

- PhenIX prioritization mode
- Autosomal Recessive inheritance mode for compound heterozygous and recessive homozygous variants
- Autosomal Dominant inheritance mode for dominant *de novo* variants
- No filter by allele frequency
- Keep only PASS values
- Variants sorted by decreasing value of the combined score (variant + gene)
- HPO terms: extracted from ClinVar annotation of the variants

Imperfect HPO phenotype generation

The algorithm we used to generate an imperfect HPO ID set, starting from the full characterization in ClinVar is the following. We altered each list of disease-associated HPO IDs by uniformly sampling between a set of alterations. Each HPO ID in the list could be substituted with:

- The same HPO ID [in this case no alteration]
- One HPO ID among the ancestors of the current HPO ID [i.e. choosing a less specific HPO ID than the true one]

- One HPO ID among the descendants of the current HPO ID [i.e. choosing a more specific HPO ID than the true one]
- One random HPO ID [something that could be unrelated to the disease]
- Nothing [in this case the HPO ID is removed

Gene-HPO association estimation algorithm:

In order to estimate the correlation of a gene with the user-defined set of phenotypes (HPO-IDs) we adapted the Maximum Information Content Ancestor (MICA) algorithm from [1]. We extended the MICA algorithm to get a finer-grained evaluation of similarities/differences among nodes of two sub-trees of the graph. With the original MICA criterion, distance from node A to all nodes from a MICA sub-tree not containing node A is the same. In this way the farther a node (HPO ID) is in the graph, the less it is considered similar. This implementation returns similarity values between 0 and 1 against a fixed reference value, eliminating the need to rescale every time the algorithm is run using different terms as in [1], and making different runs directly comparable.

In brief, we first build a graph used for calculating Gene-HPO associations in three steps:

- Build a directed acyclic graph (DAG) of HPO terms based on the information from [2].
- Define the information content (IC) of each node t_i (i.e. HPO ID) as $IC(t_i) = -\log 2(f_{t_i})$, where f_{ti} is the frequency of t_i, or any of its descendants, in the gene-HPO associations from [3]. This way, specific HPO terms associated with few genes have higher IC than HPO terms associated to a large number of genes. Parental nodes typically have lower IC than their child nodes and the root node of the DAG has $IC = 0$.
- All edges in the graph are weighted by $E_{t_1,t_2} = \left|\left(IC(t_1) - IC(t_2)\right)\right| + 1000*$

Next, we define the similarity between HPO terms as the shortest distance between their nodes $(t_{1,} t_2)$, passing through the MICA of the two nodes, rescaled by the maximum possible distance in the graph:

$$S(t_1, t_2) = 1 - \left[IC(t_1) + IC(t_2) - 2 \cdot IC\left(MICA(t_1, t_2)\right)\right] / \left(2 \cdot max_{t \in DG}(IC)\right)$$

This formula ensures a similarity of 1 when the nodes are the same, and of 0 when two nodes are as far as possible in graph. This formula also enables assignment of different similarity values to all nodes descending from $MICA(a,b)$, with similarity decreasing when node *b* gets more specific.

Finally, we estimate the association between a gene G, and a disease phenotype set D, as:

$$\mathrm{sim}(Q \rightarrow D) = avg\left(\sum_{t_1 \in Q} max_{t_2 \in D} S(t_1, t_2)\right)$$

Where *Q* is the set of HPO terms associated to the gene G, extracted from [3].

[1] Köhler, S., Schulz, M., Krawitz, P., Bauer, S., Dölken, S., Ott, C., Mundlos, C., Horn, D., Mundlos, S. and Robinson, P. (2018). *Clinical Diagnostics in Human Genetics with Semantic Similarity Searches in Ontologies*. Am J Hum Genet. 2009 Oct 9; 85(4): 457–464.

[2] http://purl.obolibrary.org/obo/hp.obo

[3] http://compbio.charite.de/jenkins/job/hpo.annotations.monthly/lastStableBuild/artifact/annotation/ALL_SOURCES_ALL_FREQUENCIES_genes_to_phenotype.txt

* The weight correction value 1000 was chosen to statistically ensure the passage through the MICA node when using an approximation (heuristic) algorithm for calculating the path between two nodes. In brief, we applied an optimized shortest-path algorithm, which needs to ignore the directionality of nodes to work properly, and does not guarantee the passing through MICA. We tested the passage of MICA empirically on 1’000’000 random node pairs. Using a weight correction value of 1000 we always obtained the same path as expected by the exact algorithm, meaning that we expect a maximal error rate of 1e-6. Without the shortest-path algorithm, the computation time would increase approximately one thousand fold.

Supplemental Figures

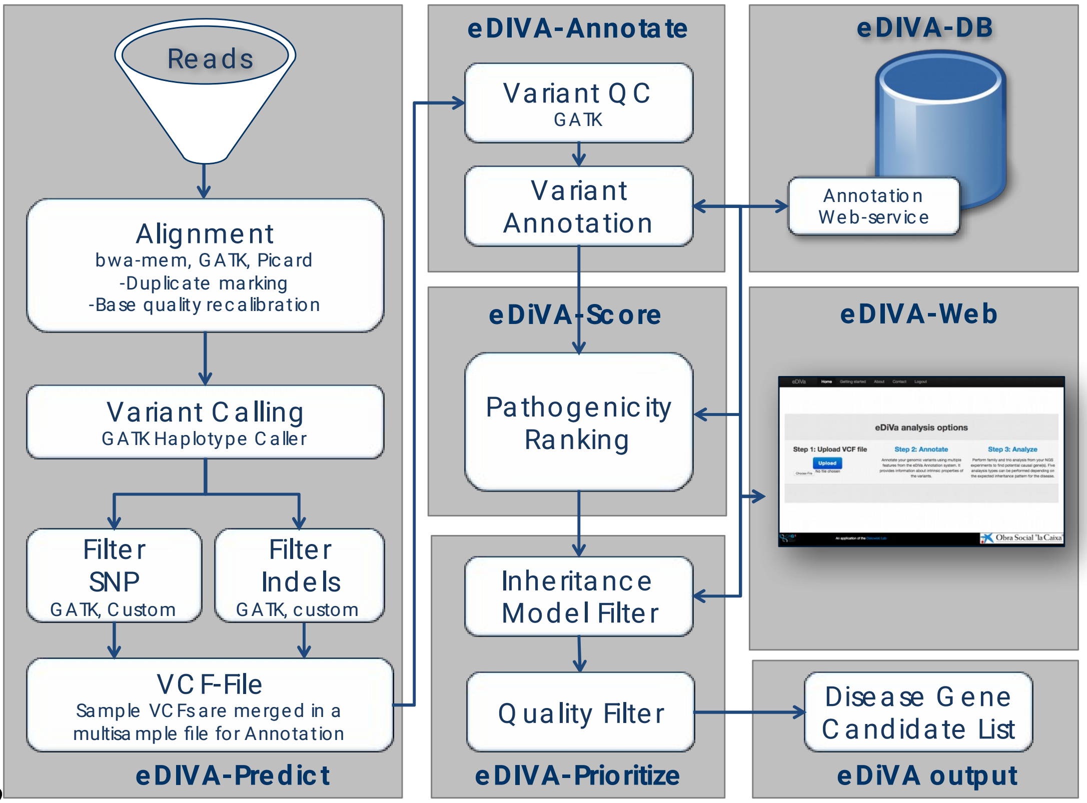

Supplemental Figure 1: eDiVa flowchart showing data processing from Fastq files to causal variant lists, including read alignment, variant calling, variant annotation, pathogenicity classification, causal variant prioritization, and output generation. eDiVA is available as stand-alone software or as a web service.

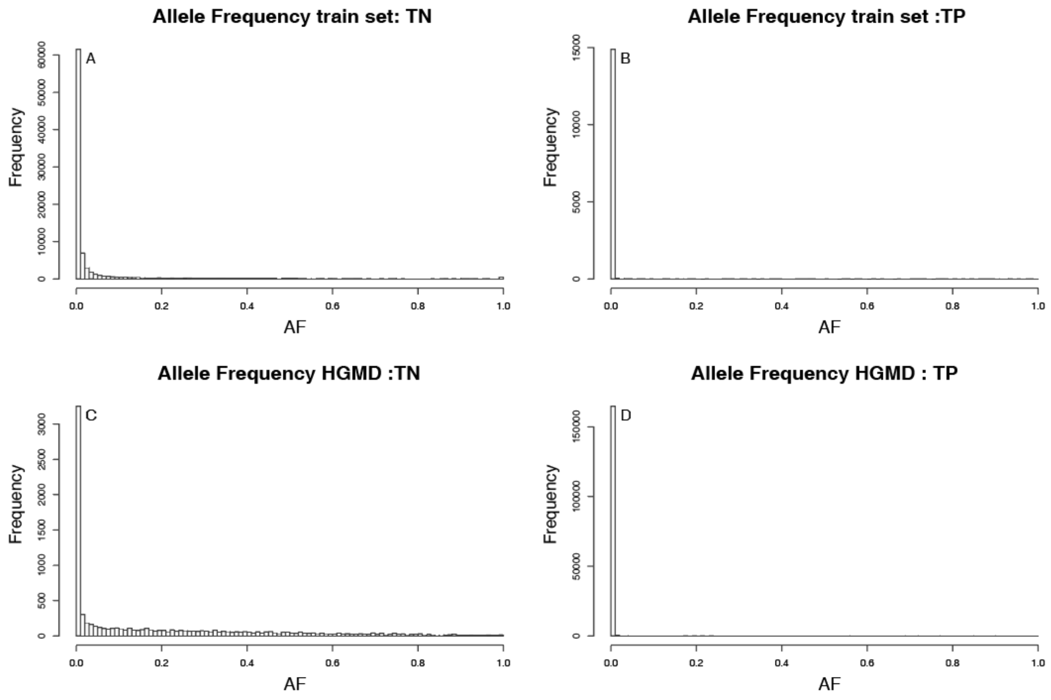

Supplemental Figure 2: Allele frequency distribution for variants used for training and benchmarking. A) AF of variants in (TN) negative training set (including ClinVar 'benign' and random GnomAD variants), B) AF of variants in (TP) positive training set (including ClinVar 'pathogenic' variants), C) AF of HGMD variants not labeled 'DM' or 'DM?', and D) AF of HGMD variants labeled 'DM' or 'DM?'.

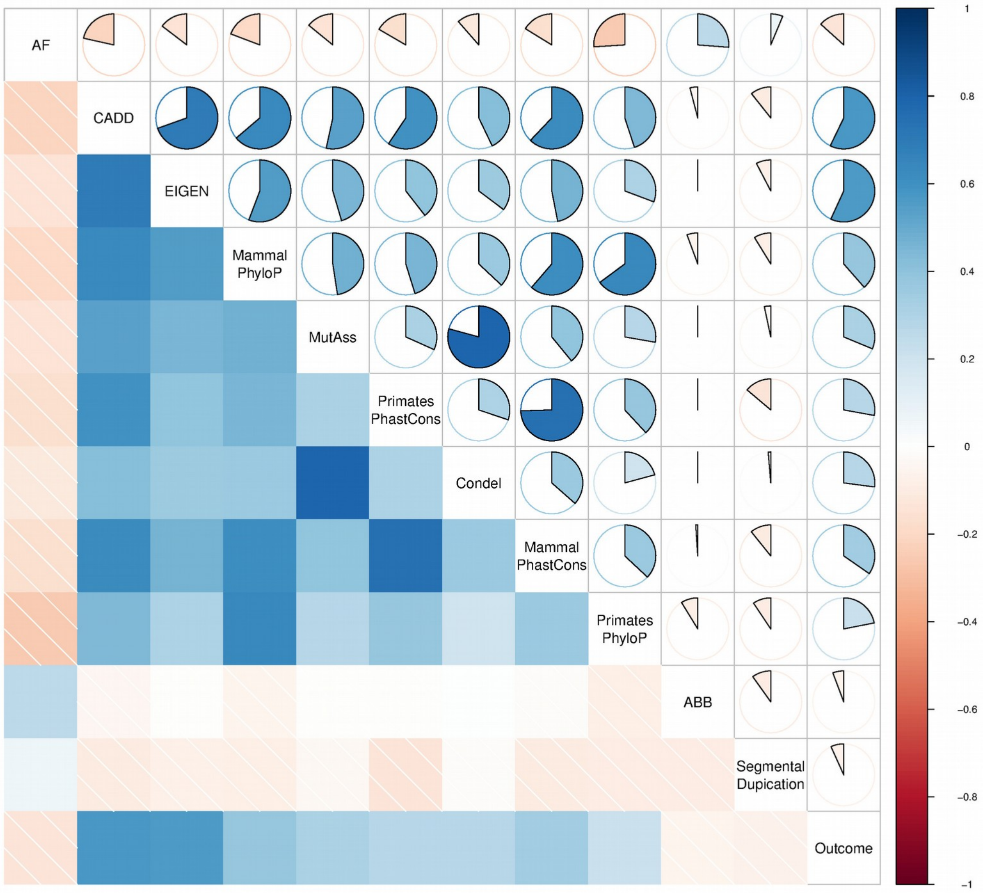

Supplemental Figure 3: Correlation matrix for features used to train the eDiVA-Score model with each other and with the outcome (correct labelling of TPs vs. TNs). Strong positive correlation is indicated by dark blue (and fraction of pie chart fill-in), while strong negative correlations are indicated by dark red (and fraction of pie chart fill-in). Strong positive correlation (although < 0.8) is observed only for MutationAssessor and Condel, for PhastCons Primates and Mammals, as well as for PhastCons and PhyloP. As expected no strong negative correlation between features is found.

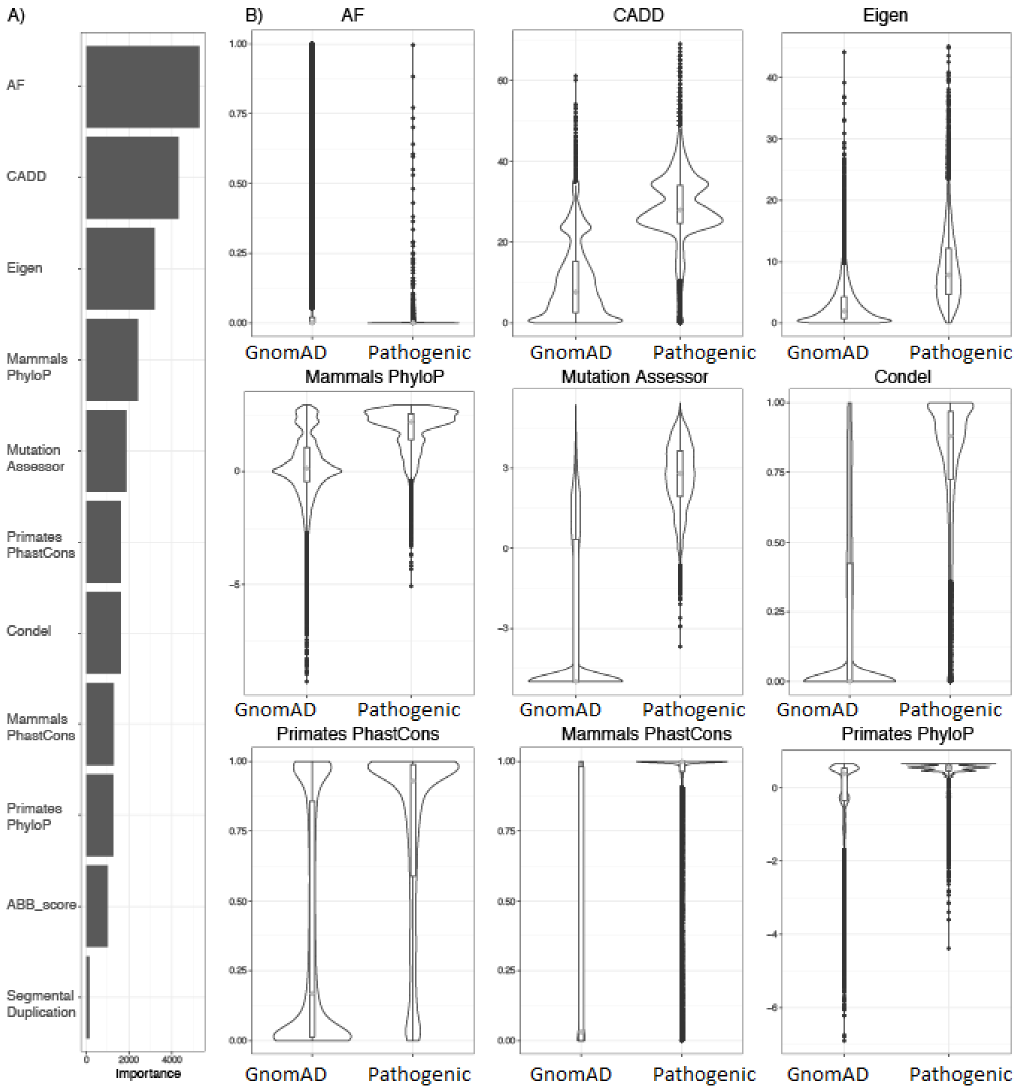

Supplemental Figure 4: eDiVA-Score random forest model: A): estimated importance of features used in the model (extracted with varImp command), and B): distribution of values for top-9 features used in the model, comparing pathogenic variants from ClinVar against random GnomAD variants.

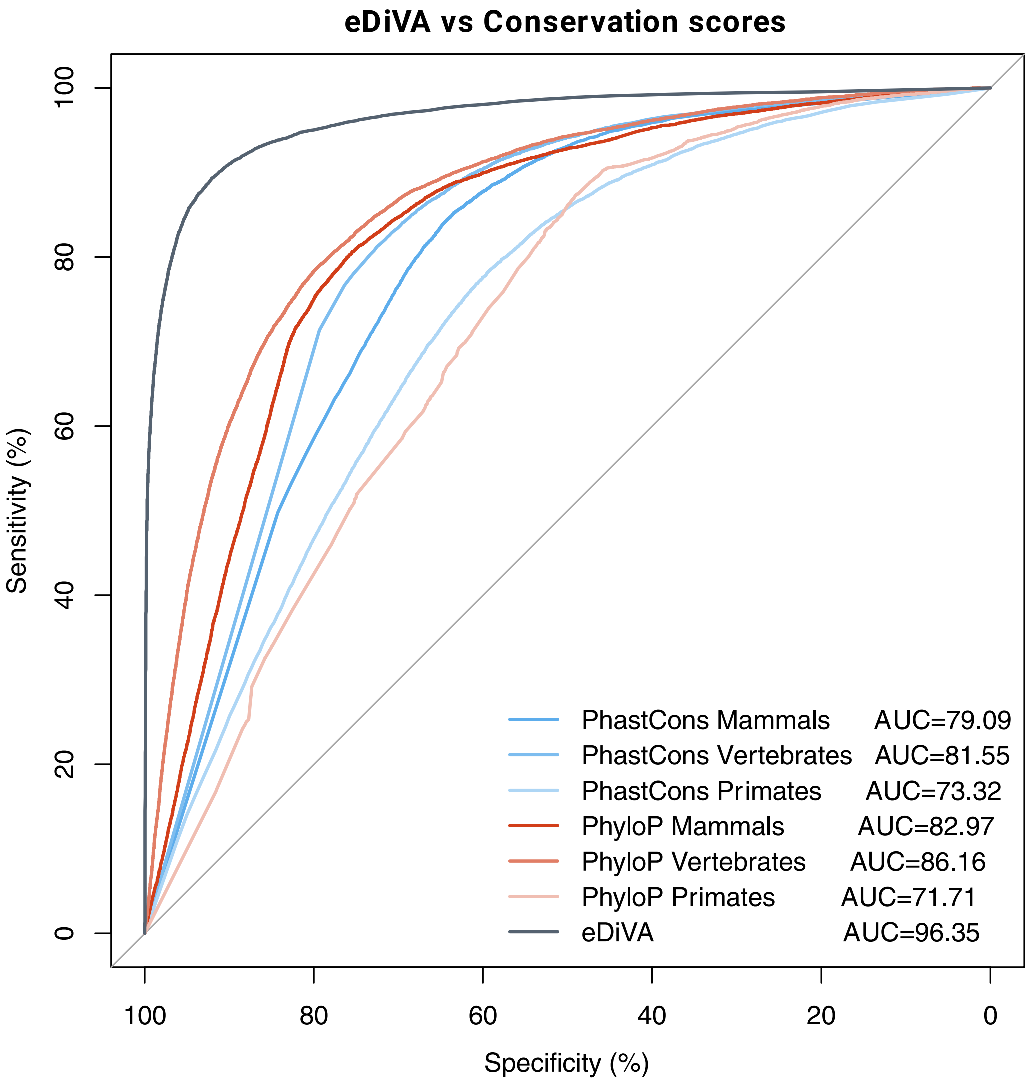

Supplemental Fig. 5 : ROC curve on independent variants from HGMD (DM and DM? as pathogenic) and 100k variants from GnomAD as benign comparing eDiVA-Score against all six conservation scores annotated by eDiVA. We found that conservation itself is a good predictor, but integration of different sources of information leads to substantially improved results.

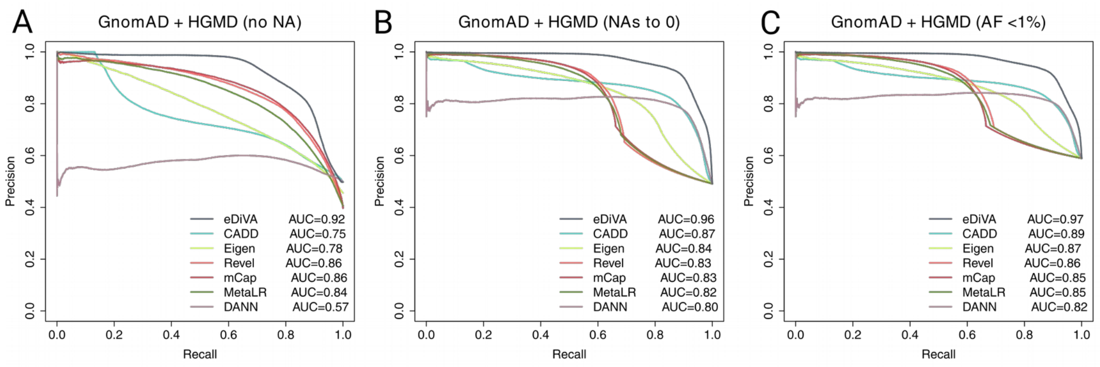

*Supplemental Figure 6: Benchmarking of pathogenicity classifiers, Precision-Recall curves on A) set of 63,712 variants from HGMD (TP) and 100,000 from GnomAD (TN) where all tools provided a prediction value B) set of 96,569 variants from HGMD (TP) and 100,000 from GnomAD (TN) after setting missing prediction values to 0, C) subset of rare variants (AF<1%) from set **B.***

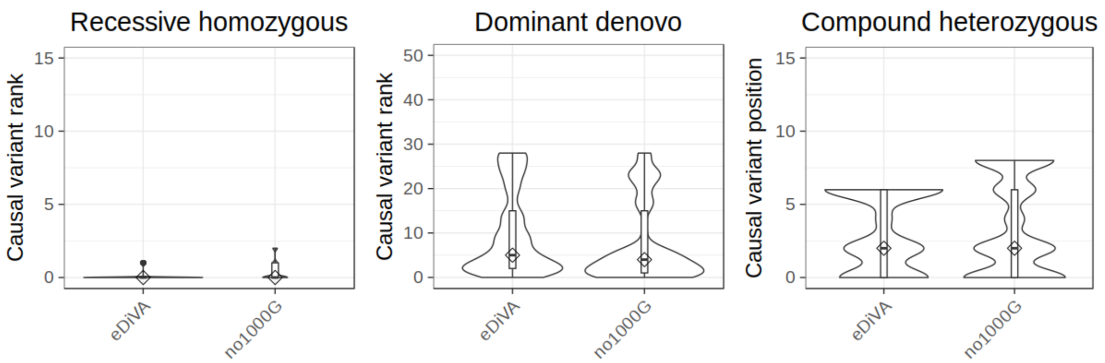

Supplemental Figure 7: Violin plot of the trio-simulations to evaluate the impact of 1000GP information on eDiVA results. The rank distribution is only mildly affected by the lack of population AF information from 1000Genomes, demonstrating that the eDiVA model is not overfitted to AF information from 1000GP.

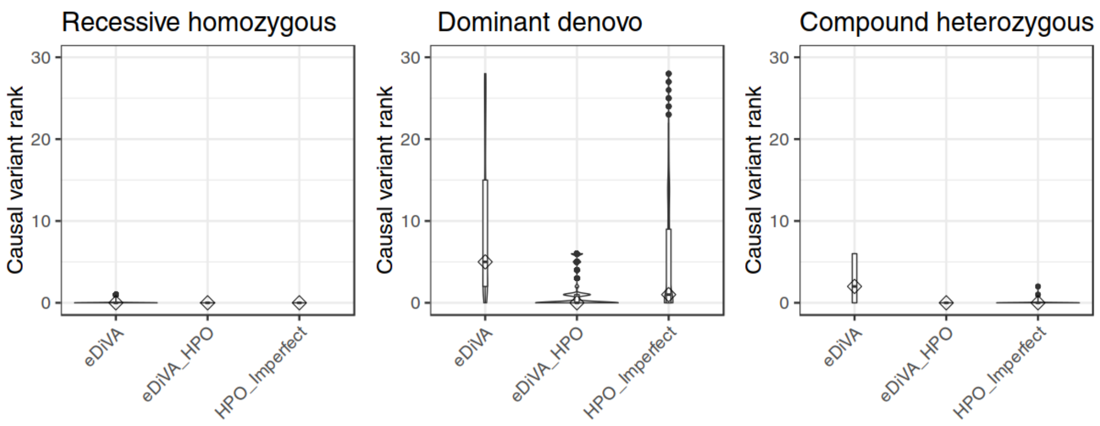

Supplemental Figure 8 : Violin plot of the trio-simulations to evaluate the impact of incomplete and imperfect phenotyping (HPO term annotation) on eDiVA's performance. We altered the complet set of HPO IDs of a gene obtained from ClinVar by randomly choosing for each HPO ID among the options i/ keep HPO ID, ii/ remove HPO ID, iii/ choose a random HPO ID, iv/ choose a random ancestor in the HPO ontology, v/ choose a random descendant in the HPO ontology. We compared the performances of eDiVA without HPO annotation ***(eDiVA)****, eDiVA with complete HPO annotation,* ***(eDiVA_HPO)*** *and eDiVA with incomplete/imperfect HPO annotation (***HPO_imperfect***). We found that an incomplete HPO description negatively affects the ranking of causal genes, but is still superior to prioritization without phenotypic information.*

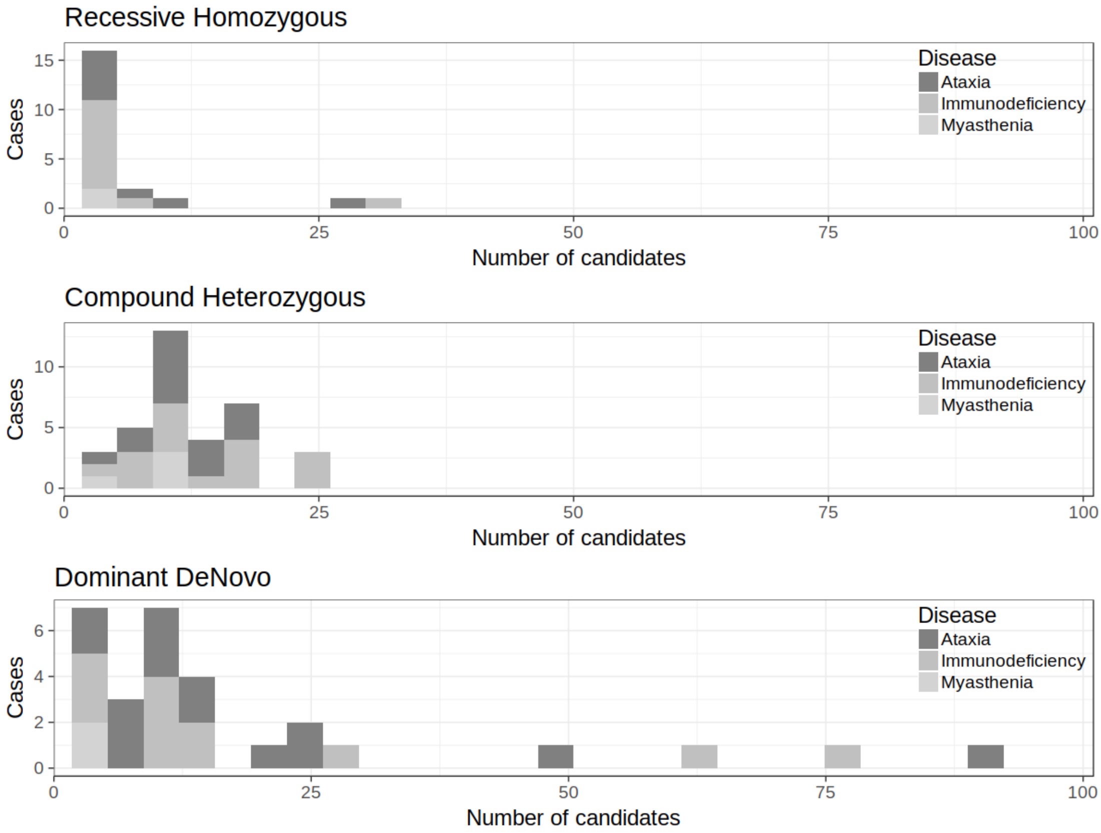

Supplemental Figure 9: Distribution of the number of candidate genes reported by eDiVA for 35 parent-child trios affected by rare diseases. Results are plotted separately by inheritance type and colored by the studied disease (i.e. Ataxia, Immunodeficiency, Myasthenia). In more than 90% of the cases eDiVA reports less than 30 candidate variants. For recessive homozygous and dominant de novo inheritance only one to five candidates are reported in the majority of cases. Outliers in dominant de novo inheritance mode are typically caused by low quality or low coverage WES data for one of the parents.

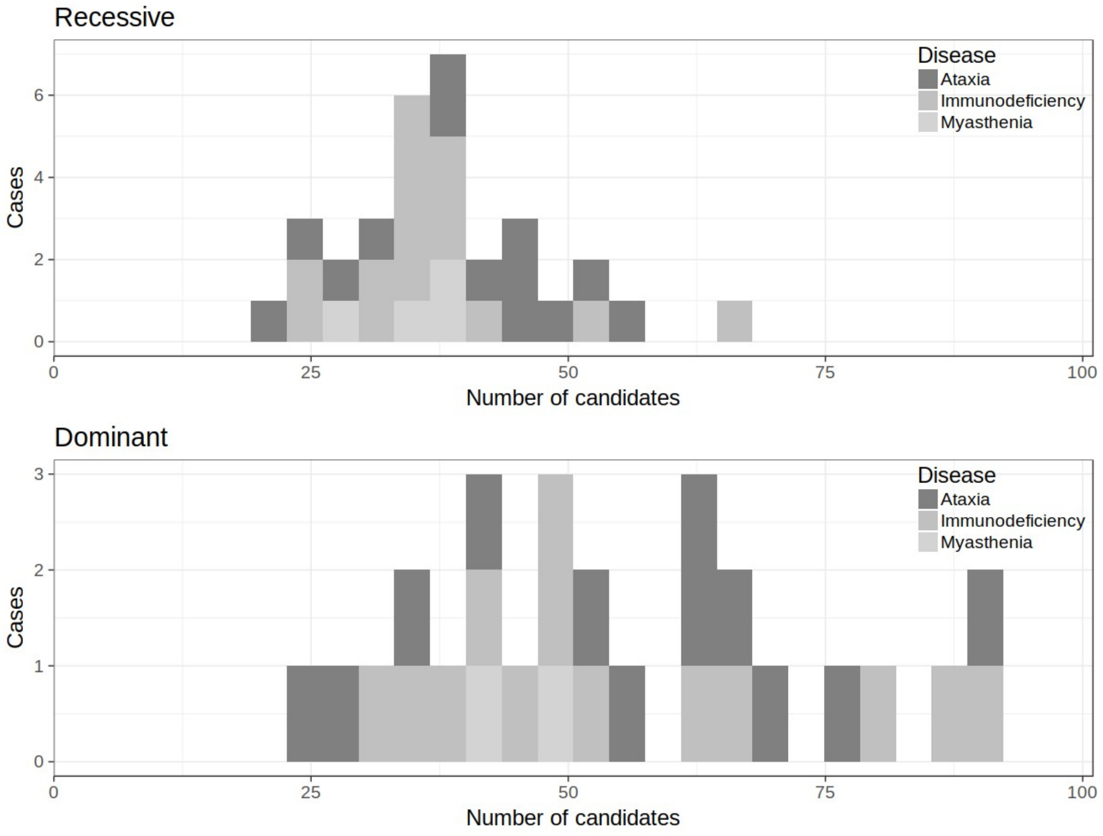

Supplemental Figure 10: Distribution of the number of candidate variants reported by Phen-Gen for 35 parent-child trios affected by rare diseases. Results are plotted separately by inheritance type and colored by the studied disease (i.e. Ataxia, Immunodeficiency, Myasthenia). Phen-Gen reports a median of 36 candidate genes for recessive and a median of 52 candidate genes for dominant inheritance modes.

Supplemental Tables

Supplemental Table 1: Default variant filter parameters of eDiVA used for WES analysis. Parameters for inheritance modes supported for parent-child trios differ in maximum population AF threshold, the zygosity requirements for each sample, and the minimum CADD score.

<table>
<tr><th>Filter</th><th colspan="3">Recessive homozygous</th><th colspan="3">Dominant de novo</th><th colspan="3">Compound heterozygous</th></tr>
<tr><td>Maximum variant frequency in healthy population</td><td colspan="3">3%</td><td colspan="3">1%</td><td colspan="3">2%</td></tr>
<tr><td>Exonic or splicing function</td><td colspan="3">X</td><td colspan="3">X</td><td colspan="3">X</td></tr>
<tr><td>Exclude if synonymous SNV</td><td colspan="3">X</td><td colspan="3">X</td><td colspan="3">X</td></tr>
<tr><td>Exclude if unknown amino acid change</td><td colspan="3">X</td><td colspan="3">X</td><td colspan="3">X</td></tr>
<tr><td>Exclude if segmental duplication > 0</td><td colspan="3">X</td><td colspan="3">X</td><td colspan="3">X</td></tr>
<tr><td>CADD</td><td colspan="3">>=0</td><td colspan="3">>19</td><td colspan="3">>=0</td></tr>
<tr><td rowspan="2">Zygosity requirements
Child | Parent | Parent</td><td>1/1</td><td>0/1</td><td>0/1</td><td>0/1</td><td>0/0</td><td>0/0</td><td>0/1</td><td>0/0</td><td>0/1</td></tr>
<tr><td colspan="6"></td><td>0/1</td><td>0/1</td><td>0/0</td></tr>
</table>

Supplemental Table 2: Number of semisynthetic cases per inheritance type simulated for benchmarking of disease variant prioritization methods. Pathogenic variants obtained from ClinVar have been integrated in WES data of a parent child trio (CEPH family from Coriell) to obtain a total of 6811 cases for which phenotypic information was available in form of HPO terms. Genotypes for each inheritance mode are shown.

Inheritance	Number of cases	Simulated genotypes		
		NA21891	NA12892	NA12878
Homozygous recessive	3353	0/1	0/1	1/1
Dominant de-novo	2592	0/0	0/0	0/1
Compound heterozygous[1]	866	0/1<br>0/0	0/0<br>0/1	0/1<br>0/1

[1] Each compound pair is composed of two variants located in the same gene with a distance grater than three base pairs.

Getting started using the eDiVA platform:

Create login at www.ediva.crg.eu

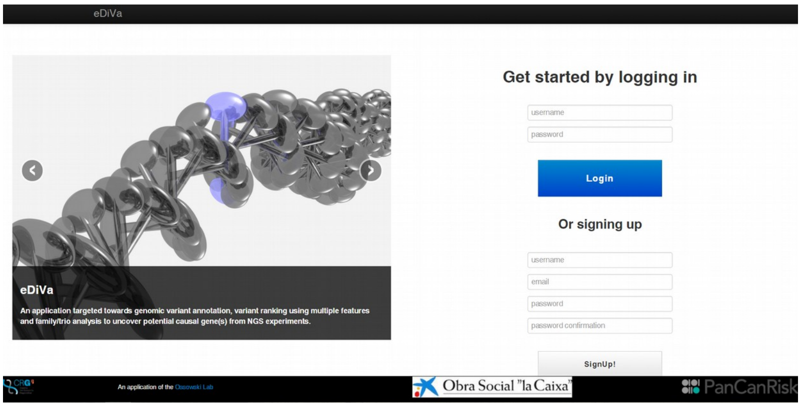

To create a login, please specify a valid email address and choose a user name and password. The account will be active immediately after signup and can be used to login to eDiVA. Alternatively, a guest user is available for testing purposes. Data in the guest user workspace may be deleted without warning. The guest account is not intended for performing analysis on access-restricted data, as any other user can access the results.

Guest account:

username: guest

password: ediva_test

eDiVA Analysis.

eDiVA’s causal variant analysis consists of three steps:

1) Uploading the variant file in VCF format,

2) Functional annotation of the variants and ranking by eDiVA’s pathogenicity score

3) Prioritization using segregation and clinical information (phenotypes).

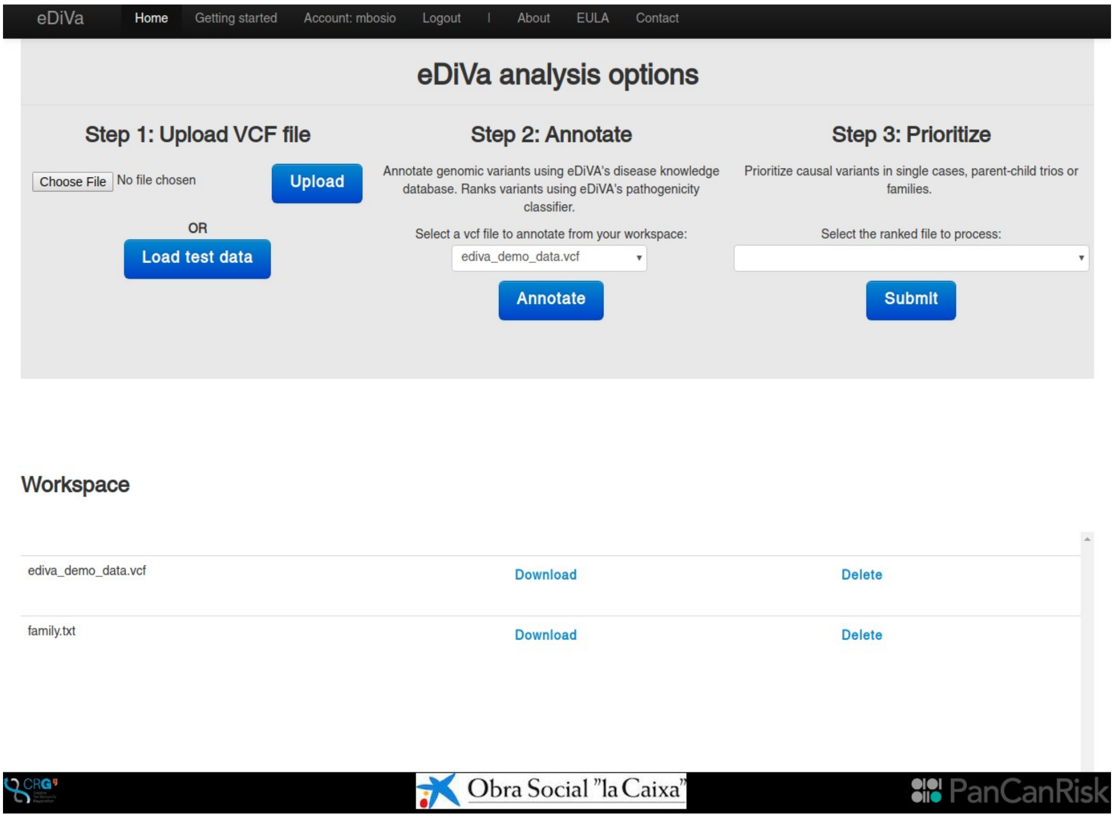

After logging into eDiVa, start with uploading the VCF file containing variants for a single case, a parent-child trio or a larger family. Trio and family variants need to be provided as multi-sample VCF file. The uploaded files will appear in the workspace section, which occupies the lower half of the browser page. The workspace will also contain all result files generated by eDiVA.

Second, select the VCF file in drop down menu of the Step 2: Annotate section and press the Run button. The annotation step will require a few minutes to compute and an email is sent once the step is finished. You can also press the reload button of the browser after a few minutes and the annotated variant file should appear in the workspace section.

Third, select the annotated variant file in the drop down menu of the Step 3: Prioritize section and press the Submit button. Pressing the Submit button of Step 3 will load a new page for causal variant prioritization analysis.

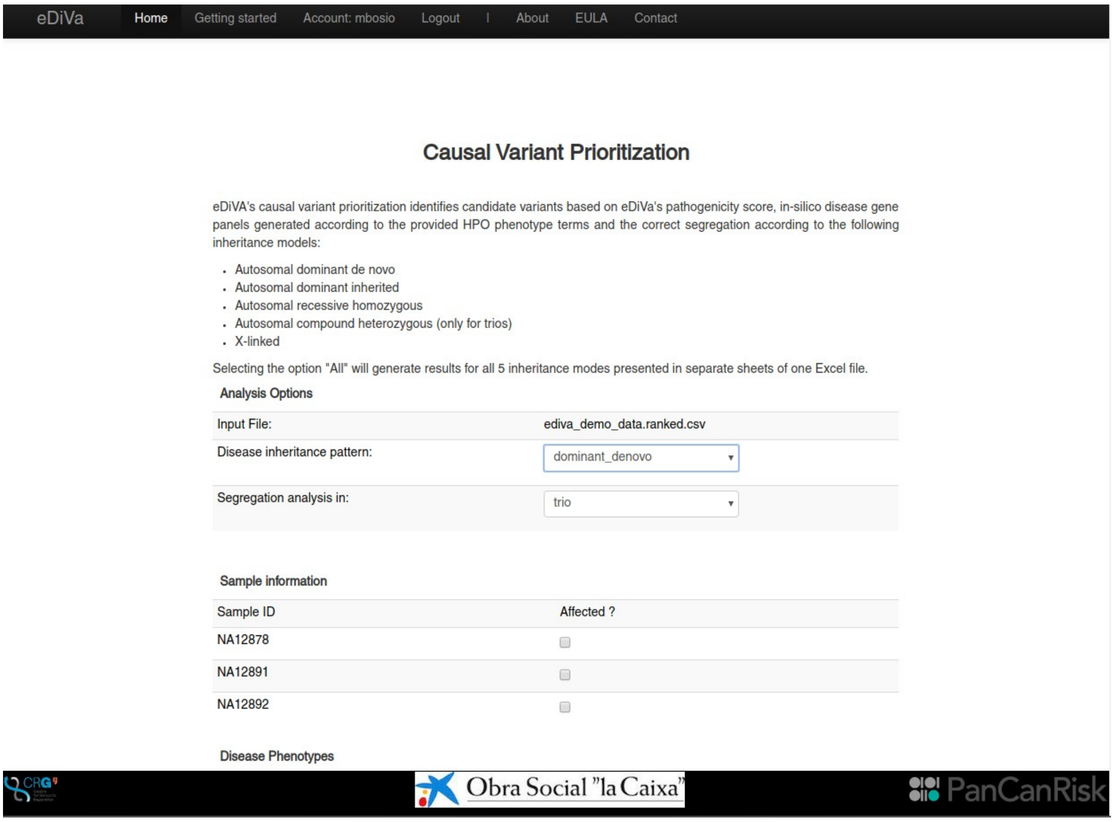

Here, select the inheritance type for your experiment, or select 'all' for running all possible analyses in one go. The following inheritance modes are supported:

- Dominant_denovo
- Dominant_inherited
- Recessive
- Xlinked
- Compound

Second, select the type of segregation analysis (single case, trio or family) and select the samples that are affected by the disease. Finally, please use the text box to specify the disease phenotypes in form of HPO terms (one HPO ID per line). Follow the link next to the text box to use the HPO term search interface to obtain suitable HPO terms.

Finally, genes can be excluded from causal variant prioritization by selecting the predefined blacklist containing genes frequently appearing as false positives (i.e. genes that appeared as incidental findings in many studies of different diseases). In addition a custom blacklist of genes can be defined, which will also be excluded. Press the submit button to start the analysis. This will bring the user back to eDiVA's workspace page, were the result file of the prioritization will be found after a few minutes of computation. An email is sent to the user once the computation has been finished.

The analysis results will appear in the workspace as a .zip file containing all processed data. The main result file is "variant_prioritization_report.xlsx" which is an excel spreadsheet containing all candidate variants organized by inheritance type (e.g. one sheet per inheritance type). The zipped file also contains the intermediate analysis files in csv format containing unfiltered annotated variants, which are useful in case no suitable candidate gene is found in the excel file. For each inheritance type there are two main files and a result log file:

- filtered .{inheritance}.csv : containing the candidate variants for {inheritance}
- unfiltered.{inheritance}.csv: containing all annotated variants, specifying for each variant the reason for being excluded or included, the HPO relatedness score, and the final ranking (i.e. columns: inheritance, filtered, HPO_relatedness, final rank columns).

- .job.log : containing the execution log file for the prioritization process.

Example case

eDiVA comes with an example case for quickly testing the tool with a few clicks. On the homepage please click the button “Load test data” to populate your workspace with a multi-sample VCF file.

Next, please follow the instructions for variant annotation (step 2) and variant prioritization (step 3) as described above.

In order to test the prioritization algorithm, we used a healthy trio (CEPH) and spiked in causal disease mutations from ClinVar for three inheritance types: recessive homozygous (Biotinidase deficiency), dominant de novo, (Pallister-Hall syndrome), and compound heterozygous (Familial hypokalemia-hypomagnesemia). In step 3 (prioritization) please use the following subset of the ClinVar reported HPO terms for the respective disease and inheritance types you wish to test:

- Recessive homozygous: HP:0000407, HP:0000572, HP:0000648, HP:0001051, HP:0001250, HP:0001251, HP:0001252, HP:0001263, HP:0001987, HP:0002014, HP:0002240, HP:0002506, HP:0008872

- Dominant de novo: HP:0000028, HP:0000110, HP:0001360, HP:0001511, HP:0004322, HP:0012165

- Compound heterozygous: HP:0000128, HP:0000848, HP:0000934, HP:0001250, HP:0002027, HP:0002900, HP:0002917, HP:0003127, HP:0003324, HP:0003470, HP:0005567

For each inheritance type you can run the prioritization analysis as explained before, specifying NA12878 as affected and NA12891 and NA 12892 as unaffected. Please include the relevant HPO terms in the text field (one HPO ID per line) to run the analysis.
